# Luminance-Degradation Compensation Based on Multistream Self-Attention to Address Thin-Film Transistor-Organic Light Emitting Diode Burn-In

**DOI:** 10.3390/s21093182

**Published:** 2021-05-03

**Authors:** Seong-Chel Park, Kwan-Ho Park, Joon-Hyuk Chang

**Affiliations:** Department of Electronics and Computer Engineering, Hanyang University, Seoul 04763, Korea; psc0902@hanyang.ac.kr (S.-C.P.); rhksgh7370@naver.com (K.-H.P.)

**Keywords:** thin-film transistor (TFT), organic light-emitting diode (OLED), compensation circuit, luminance degradation, artificial intelligence, deep neural network, convolutional neural networks

## Abstract

We propose a deep-learning algorithm that directly compensates for luminance degradation because of the deterioration of organic light-emitting diode (OLED) devices to address the burn-in phenomenon of OLED displays. Conventional compensation circuits are encumbered by high cost of the development and manufacturing processes because of their complexity. However, given that deep-learning algorithms are typically mounted onto systems on chip (SoC), the complexity of the circuit design is reduced, and the circuit can be reused by only relearning the changed characteristics of the new pixel device. The proposed approach comprises deep-feature generation and multistream self-attention, which decipher the importance of the variables, and the correlation between burn-in-related variables. It also utilizes a deep neural network that identifies the nonlinear relationship between extracted features and luminance degradation. Thereafter, luminance degradation is estimated from burn-in-related variables, and the burn-in phenomenon can be addressed by compensating for luminance degradation. Experiment results revealed that compensation was successfully achieved within an error range of 4.56%, and demonstrated the potential of a new approach that could mitigate the burn-in phenomenon by directly compensating for pixel-level luminance deviation.

## 1. Introduction

Currently, two types of displays are widely used. The first is the liquid crystal display (LCD), which generates images by controlling the amount of light emitted by the backlight unit (BLU). The second is organic light-emitting diodes (OLEDs), which generate an image by controlling the amount of current supplied to the OLED device. OLED displays have significant advantages, such as high color-reproduction ranges, low power consumption, high brightness, high contrast ratio, and a wide viewing angle [1,2,3]. However, despite their excellent performance, they are hindered by the burn-in phenomenon that is caused by the operating mechanism of OLED displays. Its panels are composed of thin-film transistor (TFT)-OLED devices mounted on each pixel, and they function as follows. First, voltage is applied to the TFT device. Second, the TFT device controls the amount of current supplied to the OLED element according to the applied voltage. Lastly, the OLED device controls the brightness of the display by adjusting luminance according to the supplied current. In this operation, the OLED device is exposed to high temperature levels owing to its luminescence characteristics. If this situation persists, it leads to problems with the driving voltage deviation of the TFT device and luminance deviation of the OLED device. Eventually, as usage time increases, the deterioration of the OLED device accelerates, and luminance degradation occurs [4,5]. Xia et al. [6] reportedd that OLED luminance degradation is caused by intrinsic and/or extrinsic factors. Intrinsic factors are generally related to moisture or oxygen that can be the cause of electrode delamination or oxidation. Extrinsic factors are related to the degradation of supply voltage–current and the change of ambient temperature during the whole lifetime of OLED displays [7,8]. In addition, Kim et al. [9] reported the characteristics of color and luminance degradation. They used an electroluminescence (EL) degradation model of R, G, and B pixels over stress time, and the blue pixel degraded faster than other pixels do. They also found that luminance degradation tends to rapidly decrease at the beginning of use, and then become more gradual. Several studies showed power consumption for the R, G, and B components of an OLED pixel by power models. Blue consumes more power than red and green components do [10,11,12]. Ultimately, the burn-in phenomenon is a major cause of image and video-quality deterioration over time [13,14,15,16]. Therefore, research on pixel compensation technology that effectively addresses the burn-in phenomenon of OLED displays is important to continuously provide high-quality images and videos to users.

Traditionally, the compensation technology of OLED displays is typically based on two types of compensation circuits. First, the internal compensation circuit controls the driving voltage of the TFT device with pixel circuits such as 5T1C or 4T0.5C to compensate for luminance degradation. The internal circuit can compensate for deviation in the driving voltage of the TFT device. However, when an internal compensation circuit is added, the structural design requirements of the TFT-OLED device become complex, and a highly sophisticated process is required [17]. Moreover, when this internal compensation circuit is utilized, it is difficult to miniaturize the pixels. Therefore, alternative circuit-compensation methods are needed for the ultraminimization of pixels that is necessary to develop high-resolution OLED displays. Second, the external compensation circuit is a mechanism that senses the characteristics of TFT elements inside the panel using sensing circuits from the outside. It then performs a compensation operation in the data voltage application section [18,19]. That is, this circuit is composed of various types of sensing devices. However, the compensation circuit requires additional external sensing circuits, logic circuits, and external memory with a simple pixel structure. In particular, an analog-to-digital converter (ADC) is required for sensing, in addition to memory for storing the sensing data. Thus, the cost of development is higher than that of the internal compensation circuit. Therefore, more effective technology is required to design and build a low-cost and high-performance compensation circuit.

We propose a deep-learning algorithm that directly compensates for luminance degradation in real time by using a data-driven approach to address the disadvantages of internal and external compensation circuits. Deep learning is a machine-learning paradigm that infers information and extracts features from the given data using multiple processing layers. Results of several studies showed that deep learning facilitates improved performance compared to traditional approaches in a variety of applications that use sensor data [3,20,21,22,23]. In this study, usage time, temperature, average brightness, data voltage deviation of the TFT device, and current supplied from TFT to OLED were used as input data for training the proposed deep-learning algorithm. In addition, deviation between the initial luminance of the OLED device and luminance degradation due to deterioration was used as the target data. As such, target data were the luminance that was compensated, and this value was obtained by subtracting the degraded luminance value from the initial luminance. When a deep-learning algorithm is trained using input and target data composed of these variables, it performs as a novel circuit that directly estimates the luminance that requires compensation. Ultimately, limitations of the existing internal and external compensation circuits can be addressed, such as the complexity of circuit design, high cost, and the difficulty of miniaturization. In addition, when TFT-OLED devices were changed, the compensation circuit had to be redesigned according to the new characteristics. However, the proposed deep-learning algorithm can relearn and reuse the characteristics of the new TFT-OLED device. We evaluated the performance of the model by calculating the deviation between the compensated luminance and the initial luminance in frames to evaluate the performance of the proposed method, and to address the phenomenon problem, which is spread within an error range of 4.56%.

## 2. Data Simulator

A data simulator is proposed to obtain the burn-in-related variables of TFT-OLED devices, similar to a typical environment. First, the proposed data simulator used the specifications of LD420EUN-UHA1 as a Si:H transistor, which is a TFT model. Using the data simulator, pixel-by-pixel data can be generated from 0 to 10,000 h in 100 h increments from the input video. In addition, pixel data can be generated from OLED displays for low temperatures, room temperature, and high temperatures from 0 to 60 ${}^{\circ }C$. The data simulator was developed to create data similar to a typical environment by mixing white noise with the generated pixel data. The data generated in this study were used to train the deep-learning model as shown in Section 4; the deep-learning model was used to examine the correlation between the variables related to the TFT-OLED device that fluctuated in real time and the luminance of the deteriorated OLED device.

**Figure 1:**
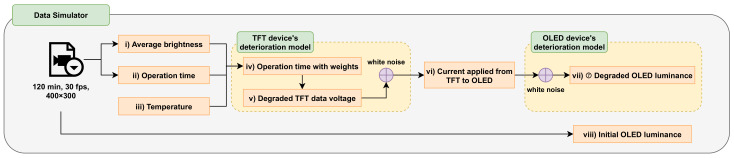
Proposed data simulator.

Figure 1 shows a block diagram of the data simulator. The input video comprised various content-specific videos with a total length of 120 min, 30 fps, and a pixel size of 400 × 300 pixels. The detailed configuration of the input videos of the data simulator is presented in Table 1. Table 2 lists the parameters used in this section.

The order of operation of the data simulator is as follows:

First, the data simulator outputs (i) the average brightness per pixel ($\bar{B_{p}}$) and (ii) operation time ($t_{p}$) from the input video. It also adds (iii) a temperature condition (*T*) between 0 and 60 ${}^{\circ }C$, which affects the deterioration of the TFT and OLED devices.
The previously obtained $\bar{B_{p}},t_{p},T$ variables are used to output (iv) the operation time with weights per pixel ($t_{p}^{\prime }$) and (v) the degraded TFT data voltage ($V_{d,t_{p}^{\prime }=\gamma }$) with the change in time and temperature. White noise is also mixed to create conditions similar to real-world environments.
$V_{d,t_{p}^{\prime }=\gamma }$ is used for each time and temperature to output (vi) the degraded OLED current ($I_{d,t_{p}^{\prime }=\gamma }$) of the TFT and to mix the white noise.
(vii) Degraded OLED luminance ($L_{t_{p}^{\prime }=\gamma }$) is observed using $I_{d,t_{p}^{\prime }=\gamma }$ for each time and temperature. (viii) The initial OLED luminance ($L_{t_{p}=0}$) is obtained directly from the input video.

Algorithm 1 shows the calculation process for the operation time and average brightness of the pixels for each frame from the video data input to the data simulator.
**Algorithm 1:** Calculation of operating time per pixel.
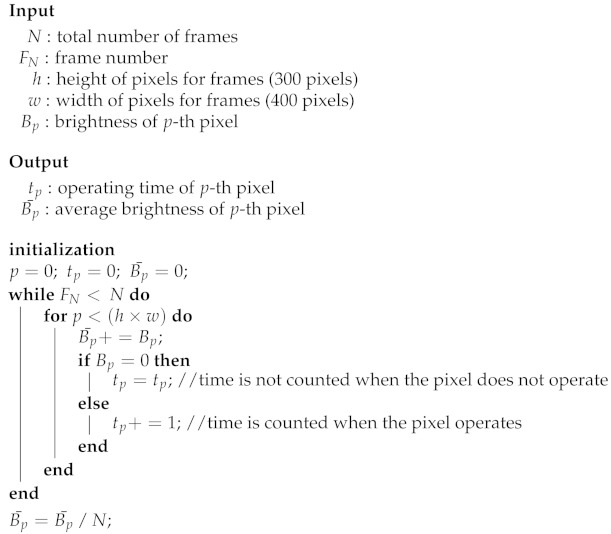


In particular, the average brightness and operation time of the input video obtained using this algorithm were used in Equation (1) to obtain the weighted operation time ($t_{p}^{\prime }$) required for the pixel to emit a specific brightness. Here, ω had a constant value of 0.8 and adjusted the weight to represent the average pixel brightness (${\bar{B}}_{p}$) during the operating time.

(1) $$t_{p}^{\prime }\;≜\;t_{p}\;\left(1+w\bar{B_{p}}\right)$$

We also generated white noise to represent the environmental noise that could occur in the OLED display using electronic circuits.

(2) $$\epsilon_{1}∼N\left(0,\frac{(\max\left(V_{th}\right)+\min\left(V_{th}\right))/2}{100}\right)$$

(3) $$\epsilon_{2}∼N\left(0,\frac{(\max(\mu )+\min(\mu ))/2}{100}\right)$$

Next, the threshold voltage shift value ($\Delta V_{shift}$), threshold voltage ($V_{th}$), and electron mobility (μ) were calculated using the previously determined $t_{p}^{\prime }$ and *T* value as follows.

(4) $$\Delta V_{shift}\;≜\;t_{p}^{\prime \;\alpha_{1}}$$

(5) $$V_{th}≜e^{\alpha_{2}\left(T-T_{\mathrm{limit}}\right)}+\;\left|\Delta V_{shift}\right|+\;\epsilon_{1}$$

(6) $$\mu ≜e^{-\alpha_{3}T}+\;\epsilon_{2}$$

The data voltage ($V_{d,t_{p}^{\prime }=\gamma }$) of the TFT was then obtained using $V_{th}$ and μ [4]. Here, $V_{\mathrm{DD}}$ was the drain voltage of 4.9 *V* and additive noise ϵ.

(7) $$V_{d,t_{p}^{\prime }=\gamma }≜V_{\mathrm{DD}}-\sqrt{\left(\frac{100}{100-\alpha })(\frac{n}{l})\frac{2I_{\max}{}^{\prime }}{\mu }C_{\mathrm{ox}}(\frac{W}{L}\right)}-|V_{\mathrm{th},t_{p}^{\prime }=\gamma }|+\epsilon$$

We used $V_{th}$ and $V_{d,t_{p}^{\prime }=\gamma }$ to determine the current applied from the TFT to the OLED ($I_{t_{p}^{\prime }=\gamma }$), such that

(8) $$I_{t_{p}^{\prime }=\gamma }≜\frac{\beta }{2}(V_{\mathrm{DD}}-V_{d,t_{p}^{\prime }=\gamma }-|V_{th}\left|\right),\;\beta =\mu C_{i}\frac{W}{L}$$

where $I_{t_{p}^{\prime }=\gamma }$ is used to obtain the luminance value ($L_{t_{p}^{\prime }=\gamma }$) at a specific time. The following is a mathematical model of the deterioration characteristics of the OLED device, where $m_{I}$ and $\eta_{I}$ have constant values [24].

(9) $$L_{t_{p}^{\prime }=\gamma }≜L_{0}exp\left\{-{\left(\frac{t_{p}^{\prime }}{\eta_{I}{\left(\frac{I_{0}}{I_{t_{p}^{\prime }=\gamma }}\right)}^{\beta }}\right)}^{m_{I}}\right\}$$

When a video was served as input to the data simulator, eight variables associated with the deterioration of the TFT-OLED device were created. Four of the eight variables were used as input data for the deep-learning algorithm, and the luminance deviation obtained by subtracting the degraded OLED luminance value from the initial OLED luminance value was used as the target data. In addition, all pixels of the OLED were independently driven; therefore, the correlation between each pixel datum generated by the data simulator was not considered. As such, 6000 independent OLED burn-in data points were generated for each pixel between 0 to 10,000 h in units of 100 h and temperatures between 0 and 60 ${}^{\circ }C$ in units of 1 ${}^{\circ }C$. The total burn-in datasets that were generated were 0.12 million (400 × 300) according to the number of pixels, and 720 million datasets (100 × 60 × 400 × 300) were generated according to time and temperatures for each color: R, G, and B.

## 3. Data Augmentation

**Figure 2:**
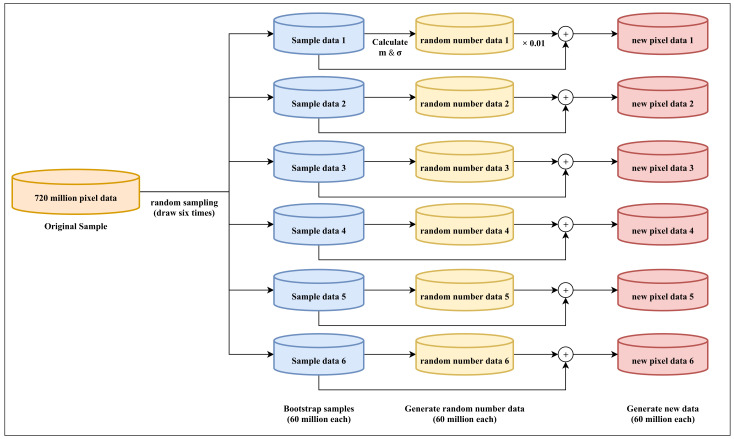
Bootstrap method.

In general, increasing the amount of data improves the performance of deep-learning models [25]. We also generated additional data via data augmentation; subsequently, we conducted training using these data with existing data. Furthermore, natural data in the real world have noise due to various conditions such as temperature, humidity, and initial tolerance. This means that it is necessary to reflect this noise and generate data similar to natural data as much as possible. The bootstrap method is an approach for increasing the training data using random sampling. Figure 2 shows a block diagram of the proposed data-augmentation algorithm based on the bootstrap method. First, 60 million samples were drawn six times using random sampling from 720 million pixel data generated through a data simulator. The extracted sample in this method is called a bootstrap sample, and the mean and standard deviation of each bootstrap sample were calculated. Then, 60 million random-number data were generated on the basis of the calculated mean and standard deviation in order to obtain noise that followed the Gaussian statistical distribution of the bootstrap sample data. The generated random-number data were multiplied by a constant weight of 0.01 to reduce information loss of the original data that may occur when noise is applied. Lastly, 60 million bootstrap sample data were multiplied by each random-number datum to generate new data. Since this method generates noise through the distribution of each bootstrap sample, it can generate noise similar to the distribution of the original image. Consequently, by applying the 720 million pixel data generated in the data simulator, 360 million training data with independent characteristics were additionally generated for each R, G, and B color.

## 4. Deep-Learning Model

### 4.1. Data Configuration

**Figure 3:**
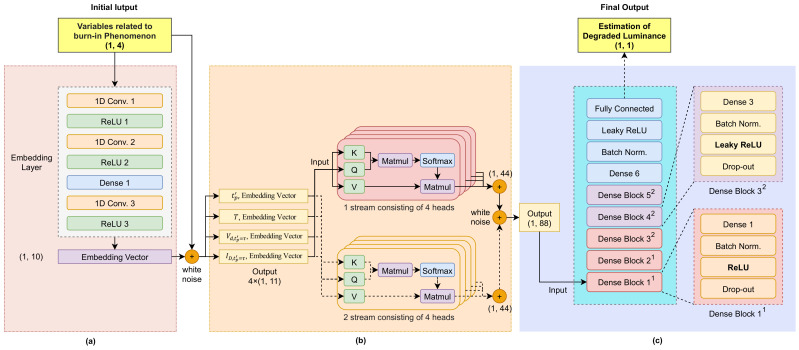
Overview of entire model: (**a**) deep feature generation; (**b**) multistream self-attention; (**c**) deep neural network.

The data used in the deep-learning model consisted of four features ($t_{p}^{\prime }$, *T*, $V_{d,t_{p}^{\prime }=\gamma }$, $I_{d,t_{p}^{\prime }=\gamma }$), consisting of vector forms with (1, 4) dimensions. The target data were one of the features, that is, deviation luminance ($L_{t_{p}=0}$ - $L_{t_{p}^{\prime }=\gamma }$) that requires compensation. The total training data consisted of 1.08 billion input data and target data pairs for each R, G, and B color. Figure 3 shows the structure of the entire proposed deep-learning model, which was trained to estimate deviation luminance (${\hat{L}}_{t_{p}^{\prime }=\gamma }$), which requires compensation.

### 4.2. Deep-Feature Generation

**Figure 4:**
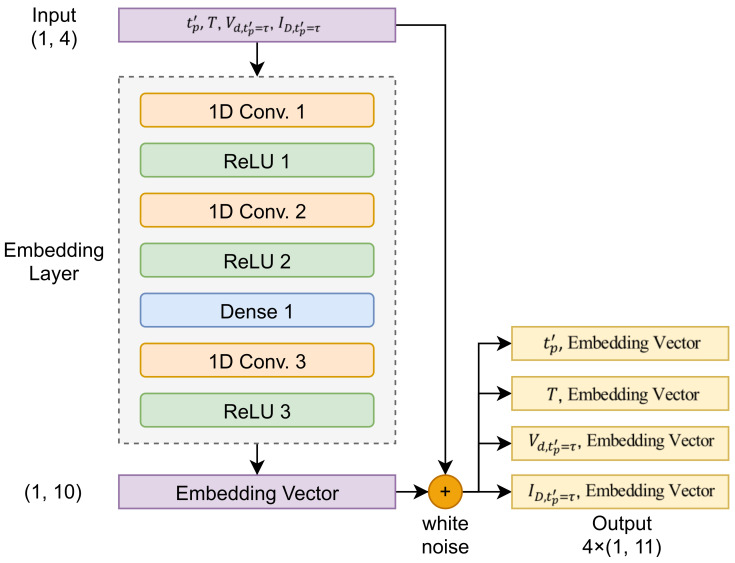
Overview of proposed deep-feature generation model.

Feature generation is also known as feature construction, feature extraction, or feature engineering. It is used to create new features from one or several features [26]. The implementation of this approach as a deep-learning technique is called deep-feature generation. Thus, in addition to features generated using the data simulator, features associated with OLED deterioration were also generated during the training process of the deep-learning model to make them similar to the OLED burn-in data. In this study, we propose a deep-feature generation algorithm composed of a 1D convolutional neural network (1D CNN), deep neural network (DNN), and rectified linear units (ReLUs) [27], which are nonlinear functions, as shown in Figure 4. Using this algorithm, new features (embedding vectors) were also extracted using existing input data with dimensions of (1, 4). First, 1D CNNs were available for 1D signal variables that could not use 2D CNNs; the computational burden is lower than that of 2D CNNs, making them suitable for real-time processing and low-cost hardware implementations [28]. In addition, a DNN extracts information on the correlation between features by completely connecting the outputs of the 1D CNN. This DNN facilitated a nonlinear combination of input features, and feature extraction was automatically performed. In the final output of this deep-feature generation algorithm, 10 new features were generated with dimensions (1, 10). White noise was mixed to represent the noise in the OLED display circuit environment. Subsequently, one of the four original features was selected and concatenated to the new features, resulting in a new form of data with a higher dimension (1, 11) than the original dimension (1, 4).

### 4.3. Multistream Self-Attention

**Figure 5:**
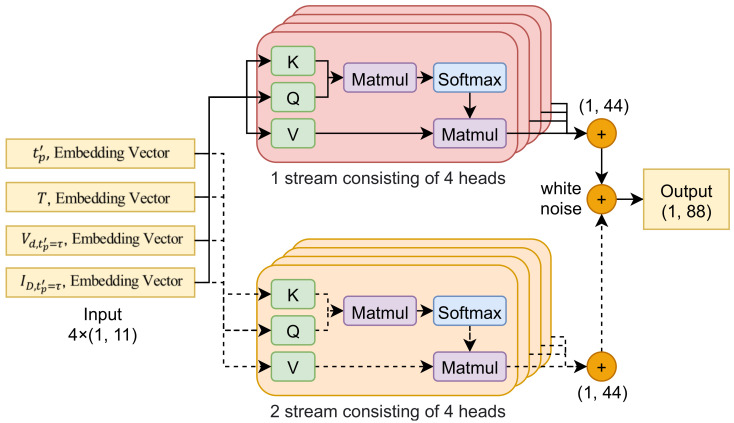
Overview of multihead self-attention model.

Multistream self-attention [29] has already been applied to the field of speech recognition. On the basis of the idea of this algorithm, we propose modified multistream self-attention that was optimized for learning the outputs of deep-feature generation. The proposed multistream self-attention consisted of two multihead self-attention layers [30], each of which consisting of four self-attention layers, as shown in Figure 5. The operation process of this algorithm proceeds in the following order. First, multistream self-attention improves the performance of deep-learning algorithms with ensemble-like effects. Second, multihead self-attention corresponding to each stream is trained by increasing the weight of the most important feature to effectively compensate for the degraded luminance among input features. Similarly, less important features are trained, such that the weight is reduced; that is, when four input data with dimensions (1, 11) are input to each head, an extraction process is performed that represents the importance of each feature by adjusting the weight value to focus on the most important of the 11 features. Third, given that the output of multihead self-attention maintains the dimension of the input data, data with the (1, 44) dimension are output by concatenating four outputs of each head. Lastly, multistream self-attention outputs data with dimensions (1, 88) as two outputs.

### 4.4. DNN

**Figure 6:**
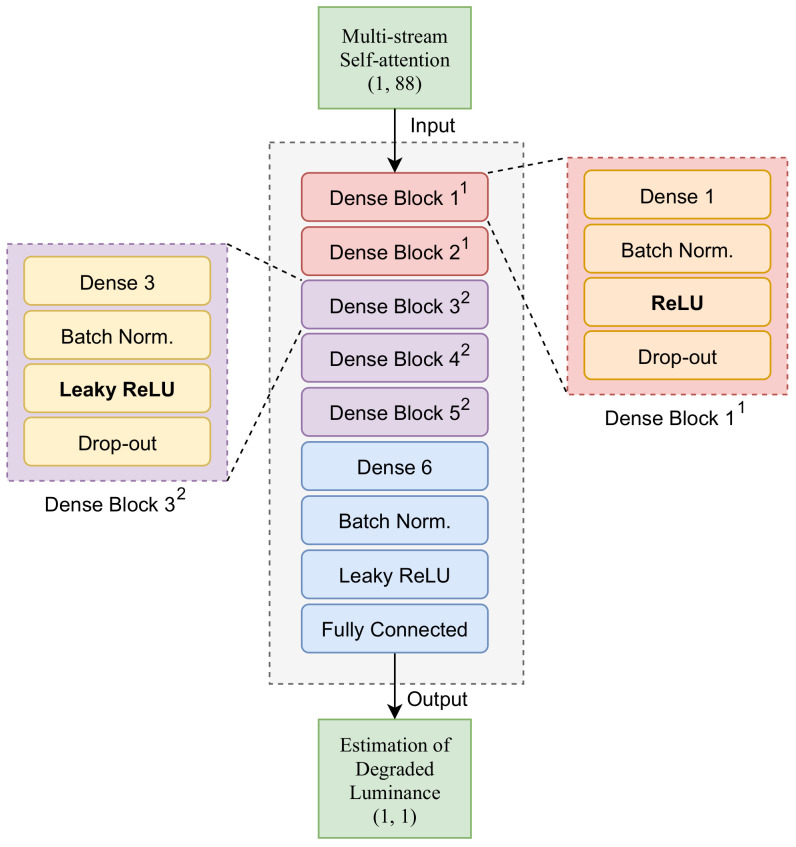
Overview of proposed deep-neural-network model.

The DNN [31] was successfully utilized in applications such as image processing, automatic speech recording, and natural-language processing. As shown in Figure 6, the proposed algorithm consisted of $DenseBlock^{1}$, $DenseBlock^{2}$, a single dense layer, and a fully connected layer. $DenseBlock^{1}$ comprised a single dense layer, batch normalization layer, ReLU, and dropout. $DenseBlock^{2}$ was a nonlinear function of $DenseBlock^{1}$, ReLU replaced by Leaky-ReLU, which is proposed to solve the dying ReLU phenomenon. This DNN algorithm was trained to identify nonlinear relationships between input data and target data by recognizing specific patterns when data with dimensions (1, 88) are input. For the final output, we obtain the value of dimension (1, 1) of luminance (${\hat{L}}_{t_{p}^{\prime }=\gamma }$) that requires compensation.

## 5. Experimental Environment and Result

### 5.1. Datasets

**Figure 7:**
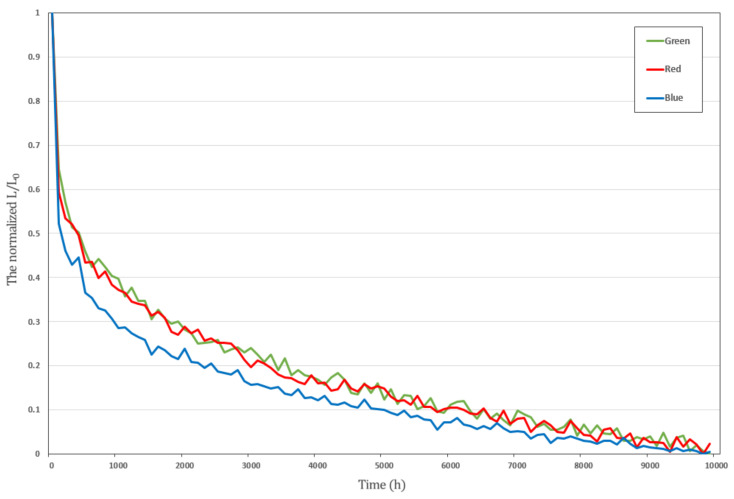
Luminance-degradation rate for normalized blue, red, and green pixel data.

In our experiments, we used blue pixel data, which have larger power consumption and much faster degradation rate than those of red and green pixel data [32]. Therefore, a deep-learning-based compensation algorithm was trained and evaluated using 1.08 billion datasets of blue pixel data generated using data simulators and data augmentation. The compositions of the datasets, divided into training data and test data, are shown in Table 3. Figure 7 shows the power consumption and luminance-degradation rate for the blue, red, and green pixels.

### 5.2. Experiment Setup

All experiments in this study were conducted using TensorFlow in the Python library. Batch normalization [33] was applied to the DNN, and the Adam optimizer [34] with a learning rate of 0.001 was used for training the deep-learning algorithm. In addition, the batch size used in the training process was 6000, and all parts of the algorithm were jointly optimized with the mean absolute percentage error (MAPE) used as a loss function in the following. The algorithm was trained for 50 epochs; if the validation loss did not improve within three epochs, an early pause was applied. In addition, the accuracy of the algorithm was calculated using MAPE, as shown below.

(10) $$\mathrm{MAPE}=\frac{1}{NP}\sum_{f=1}^{N}\sum_{p=1}^{P}\left|\frac{L_{t_{p}=0}-{\hat{L}}_{t_{p}^{\prime }=\gamma }}{L_{t_{p}=0}}\right|$$

(11) $$\mathrm{Accuracy}=100(1-\frac{1}{NP}\sum_{f=1}^{N}\sum_{p=1}^{P}|\frac{L_{t_{p}=0}-{\hat{L}}_{t_{p}^{\prime }=\gamma }}{L_{t_{p}=0}}\left|\right)\left(\%\right)$$

### 5.3. Result Analysis

As shown in Table 4, in the case of deep-feature generation, experiments were conducted with three models; Experiment 2 demonstrated the best accuracy at 91.62%.

As depicted in Table 5, based on the testing of the 1- and 2-stream self-attention algorithms, Experiment 2 showed accuracy of 92.19%. When using three or more streams, there was a tendency to overfit as the number of streams increased.

In Table 6, experiments were conducted by changing the number of DNN layers in deep neural networks; experiment 3 demonstrated an accuracy of 93.31%, which indicates that six layers are suitable.

Table 7 shows the experiment results obtained by adjusting the number of units in each layer of the DNN algorithm. The DNN algorithm used six layers, as obtained from the experiment results in Table 6. As a result, Experiment 4 with a bottleneck structure demonstrated accuracy of 95.44%.

**Figure 8:**
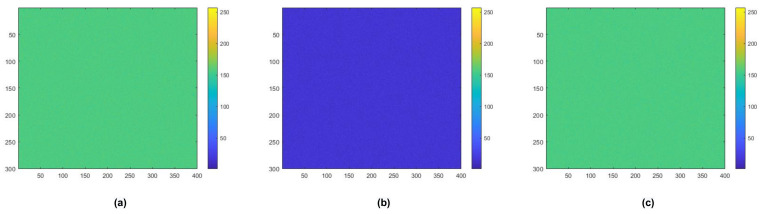
Image of OLED display (400 × 300) according to number of pixels. (**a**) Initial display image; (**b**) image in which luminance degradation occurred; (**c**) image when degraded luminance was compensated.

Therefore, as shown in Table 4, Table 5, Table 6 and Table 7, the final performance of the deep-learning-based compensation algorithm had the best accuracy of 95.44%. Figure 8 compares the initial display image, the image in which luminance degradation occurred, and the image in which the degraded luminance was compensated.

## 6. Conclusions

In this study, we proposed a deep-learning algorithm to address the burn-in phenomenon of OLED displays by using deep-learning technology. This algorithm can replace physical-circuit-based internal and external compensation circuits that compensate after sensing degraded TFT data voltage or TFT-OLED current and calculating the luminance-degradation value of OLED display due to the deterioration of OLED devices. This means that the proposed compensation method based on the deep-learning algorithm does not need to add internal and external compensation circuits for calculating luminance degradation. In particular, even if a new TFT-OLED device is developed, the significant advantage is that only the deep-learning algorithm can be relearned according to the parameters of the device and reused without the need to change the physical circuit. Furthermore, if the OLED display is combined with cloud service, the deep-learning algorithm can be easily remotely improved. In the future, we will supplement the data simulator on the basis of real data, and augment burn-in data to strengthen the deep-learning-based compensation algorithm.

## Figures and Tables

**Table 1:** Specifications of input videos.

Contents	Specifications
Content 1 (40 min)	Documentary, action, news, sports
Content 2 (40 min)	Entertainment, beauty, animation, car review
Content 3 (40 min)	Game, cooking, job introduction, romance

**Table 2:** Paper nomenclature.

Symbol	Parameter	Symbol	Parameter
$F_{N}$	Data of input video	*N*	Total frame of input video
*f*	Frame	*P*	Total pixel
*p*	Pixel	*t*	Time
$t_{p}$	Operating time per pixel	$t_{p}^{\prime }$	Weighted operating time
$B_{p}$	Brightness of per pixel	$\bar{B_{p}}$	Average brightness per pixel
$\epsilon_{1}$	Noise of threshold voltage	$\epsilon_{2}$	Noise of mobility
$\alpha_{1}$	Reduction factor of shifting value of threshold voltage	$\alpha_{2}$	Reduction factor of threshold voltage
$\alpha_{3}$	Reduction factor of mobility	$I_{\max}$	Maximum input current of TFT
*L*	Length of TFT channel	*W*	Width of TFT channel
$V_{d}^{\prime }$	Data voltage of TFT that consider noise	$V_{d,t_{p}=0}$	Initial data voltage of TFT
$C_{ox}$	Capacitor of TFT unit area	μ	Initial mobility of TFT
$V_{th}^{\prime }$	Threshold voltage of TFT that consider noise	$V_{\mathrm{DD}}$	Drain voltage of TFT
$T_{\mathrm{limit}}$	Maximal temperature of TFT performance guarantee	$\Delta V_{shift}$	Shifting value of threshold voltage
$V_{th,t_{p}=0}$	Initial threshold voltage of TFT	*w*	Weight factor
*n*	Gray level of TFT	*l*	Total gray level range
α	Reduction rate of OLED voltage	*T*	Temperature
β	Transistor parameter	$C_{i}$	Gate capacitor
*W*	Channel width		

**Table 3:** Dataset composition.

Datasets	Train/Test	Total
OLED pixel (Blue)	9.72/1.08 billion	10.8 billion

**Table 4:** Accuracy (in %) comparison of proposed models composed of different hyperparameters with deep-feature generation (layers, kernel, filter size, and units).

	Experiment 1		Experiment 2		Experiment 3	
**Experimental** **Details**	Layers	Kernel Filter Size Units	Layers	Kernel Filter Size Units	Layers	Kernel, Filter Size Units
	1D Conv 1	1 × 4 @32	1D Conv 1	1 × 4 @32	1D Conv 1	1 × 4 @32
	1D Conv 2	1 × 32 @16	1D Conv 2	1 × 32 @16	Dense 1	32
	Dense 1	16	Dense 1	16	1D Conv 2	1 × 32 @16
			1D Conv 3	1 × 16 @10	Dense 2	16
					1D Conv 3	1 × 16 @10
Accuracy	90.28%		**91.62%**		91.45%	

**Table 5:** Accuracy (in %) comparison of the proposed models with multistream self-attention [29].

Experimental Details	Experiment 1	Experiment 2
	1-Stream Self-Attention	2-Stream Self-Attention
Accuracy	90.75%	**92.19%**

**Table 6:** Accuracy (in %) comparison of proposed models with different numbers of deep-neural-network layers.

	Experiment 1		Experiment 2		Experiment 3	
	Layer Number	Units	Layer Number	Units	Layer Number	Units
**Experimental** **Details**	Dense layer 1	64	Dense layer 1	64	Dense layer 1	64
	Dense layer 2	64	Dense layer 2	64	Dense layer 2	64
	Dense layer 3	64	Dense layer 3	64	Dense layer 3	64
	Dense layer 4	64	Dense layer 4	64	Dense layer 4	64
			Dense layer 5	64	Dense layer 5	64
					Dense layer 6	64
Accuracy	89.94%		91.22%		**93.31%**	

**Table 7:** Accuracy (in %) comparison of proposed models with different numbers of units of deep-neural-network layers.

	Layer Number	Experiment 1	Experiment 2	Experiment 3	Experiment 4	Experiment 5
		Units				
**Experiment** **Details**	Dense layer 1	64	128	256	256	256
	Dense layer 2	64	128	256	128	128
	Dense layer 3	64	128	256	128	128
	Dense layer 4	64	128	256	256	256
	Dense layer 5	64	128	256	128	128
	Dense layer 6	64	128	256	64	128
Accuracy		92.58%	93.35%	93.76%	**95.44%**	95.10%

## Data Availability

Not applicable.

## References

[1] Kang S. (2016). OLED power control algorithm using optimal mapping curve determination. J. Disp. Technol..

[2] Jung H., Kim Y., Chen C., Kanicki J. (2012). A-IGZO TFT based pixel circuits for AM-OLED displays. Proc. SID Tech. Dig..

[3] Wang C., Hu Z., He X., Liao C., Zhang S. (2016). One gate diode-connected dual-gate a-IGZO TFT driven pixel circuit for active matrix organic light-emitting diode displays. IEEE Trans. Electron Devices.

[4] In H.-J., Kwon O.-K. (2009). External compensation of nonuniform electrical characteristics of thin-film transistors and degradation of OLED devices in AMOLED displays. IEEE Electron Device Lett..

[5] Lee K.-Y., Chao Y.-P., Chen W.-D. (2014). A new compensation method for emission degradation in an AMOLED display via an external algorithm, new pixel circuit, and models of prior measurements. J. Disp. Technol..

[6] Xia S.C., Kwong R.C., Adamovich V.I., Weaver M.S., Brown J.J. OLED device operational lifetime: Insights and challenges. Proceedings of the 2007 IEEE 45th Annual International Reliability Physics Symposium.

[7] Zhou X., He J., Liao L.S., Lu M., Ding X.M., Hou X.Y., Zhang X.M., He X.Q., Lee S.T. (2000). Real-time observation of temperature rise and thermal breakdown processes in organic LEDs using an IR imaging and analysis system. Adv. Mater..

[8] Kundrata J., Baric A. Electrical and thermal analysis of an OLED module. Proceedings of the Comsol Conference.

[9] Kim H., Shin H., Park J., Choi Y., Park J. Statistical modeling and reliability prediction for transient luminance degradation of flexible OLEDs. Proceedings of the 2018 IEEE International Reliability Physics Symposium (IRPS).

[10] Dong M., Choi Y.K., Zhong L. Power modeling of graphical user interfaces on OLED displays. Proceedings of the 2009 46th ACM/IEEE Design Automation Conference.

[11] Hadizadeh H. (2017). Energy-Efficient Images. IEEE Trans. Image Process..

[12] Chang T., Xu S.S. Real-time quality-on-demand energy-saving schemes for OLED-based displays. Proceedings of the 2013 IEEE International Symposium on Industrial Electronics.

[13] Scholz S., Kondakov D., Lussem B., Leo K. (2015). Degradation mechanisms and reactions in organic light-emitting devices. Chem. Rev..

[14] Langlois E., Wang D., Shen J. (2000). Degradation mechanisms in organic light emitting diodes. Synth. Met..

[15] Schmidbauer S., Hohenleutner A., Konig B. (2013). Chemical Degradation in Organic Light-Emitting Devices: Mechanisms and Implications for the Design of New Materials. Adv. Mater..

[16] Fery C., Racine B., Vaufrey D., Doyeux H., Cina S. (2005). Physical mechanism responsible for the stretched exponential decay behavior of aging organic light-emitting diodes. Appl. Phys. Lett..

[17] Lee K.-Y., Hsu Y.-P., Chao C.-P. (2012). A new 4T0.C AMOLED pixel circuit with reverse bias to alleviate OLED degradation. IEEE Electron Device Lett..

[18] Lin C.-L., Chen Y.-C. (2007). A novel LTPS-TFT pixel circuit compensating for TFT threshold-voltage shift and OLED degradation for AMOLED. IEEE Electron Device Lett..

[19] Lee K.-Y., Chao C.-P. (2012). A new AMOLED pixel circuit with pulsed drive and reverse bias to alleviate OLED degradation. IEEE Electron Device Lett..

[20] Zeng M., Nguyen L.T., Yu B., Mengshoel O.J., Zhu J., Wu P., Zhang J. Convolutional Neural Networks for human activity recognition using mobile sensors. Proceedings of the 2014 6th International Conference on Mobile Computing, Applications and Services.

[21] Ravi D., Wong C., Lo B., Yang G.-Z. (2017). A Deep Learning Approach to on-Node Sensor Data Analytics for Mobile or Wearable Devices. IEEE J. Biomed. Health Inform..

[22] Krizhevsky A., Sutskever I., Hinton G.E. (2012). Imagenet classification with deep convolutional neural networks. Adv. Neural Inf. Process. Syst..

[23] Psuj G. (2018). Degradation Mechanisms in Organic Light Emitting Diodes. Sensors.

[24] Zhang J., Li W., Cheng G., Chen X., Wu H., Shen H. (2014). Life prediction of OLED for constant-stress accelerated degradation tests using luminance decaying model. Appl. Phys. Lett..

[25] Taylor L., Nitschke G. (2017). Improving Deep Learning using Generic Data Augmentation. arXiv.

[26] Bosch S.V.D. (2017). Automatic Feature Generation and Selection in Predictive Analytics Solutions. Master’s Thesis.

[27] Agarap F. (2018). Deep learning using rectified linear units (relu). arXiv.

[28] Kiranyaz S. 1-D Convolutional Neural Networks for Signal Processing Applications. Proceedings of the ICASSP 2019—2019 IEEE International Conference on Acoustics, Speech and Signal Processing (ICASSP).

[29] Han K.J., Prieto R. (2019). State-of-the-art speech recognitions using multi-stream self-attention with dilated 1D convolutions. arXiv.

[30] Vaswani A., Shazeer N., Parmar N., Uszkoreit J., Jones L., Gomez A.N., Kaiser Ł., Polosukhin I. Attention is all you need. Proceedings of the Advances in Neural Information Processing Systems 30 (NIPS 2017).

[31] Heaton J., Goodfellow I., Bengio Y., Courville A. (2018). Deep learning. Genet. Program. Evolvable Mach..

[32] Yeh C.-H. (2019). Visual-Attention-Based Pixel Dimming Technique for OLED Displays of Mobile Devices. IEEE Trans. Ind. Electron..

[33] Ioffe S., Szegedy C. Batch Normalization: Accelerating Deep Network Training by Reducing Internal Covariate Shift. https://arxiv.org/pdf/1502.03167.pdf.

[34] Kingma D.P., Ba J. Adam: A Method for Stochastic Optimization. https://arxiv.org/pdf/1412.6980.pdf.

